# The Law of the State of Virginia Regulating the Practice of Dentistry

**Published:** 1890-06

**Authors:** 


### Editorial, Etc.

THE LAW OF THE STATE OF VIRGINIA REGULATING THE PRACTICE OF DENTISTRY.—The law regulating the practice of dentistry in Virginia, being Chapter LXXIX, Code of 1887, amended and approved January 28, 1890, (amendments in italics,) is as follows:

SECTION 1767. Who may Practice Dentistry.—From and after the passage of this act it shall be unlawful for any person to engage in the practice of dentistry in *the Commonwealth of Virginia, or to assist in the practice of dentistry as either assistant or employee*, or to receive license from any commissioner of revenue, unless such person has graduated and received a diploma from the faculty of a reputable institution where this specialty is taught, and chartered under the authority of some one of the United States, or of a foreign government, *acknowledged as such, and* shall have obtained a certificate from the board of examiners duly appointed under the provisions of *section one thousand seven hundred and sixty-eight of Code of eighteen hundred and eighty-seven*, to issue such certificates; provided, *that persons who shall be engaged in the practice of dentistry in the Commonwealth of Virginia on the first day of January, eighteen hundred and ninety, and who shall comply with the requirements of section one thousand seven hundred and seventy-four of this act shall be otherwise exempt from the provisions of this section; and provided further, that nothing contained in this section shall prevent a student who is pursuing a regular course of instruction from assisting a person in the practice of dentistry qualified as herein provided*, or shall prevent any *authorized physician or surgeon* from extracting teeth for any one suffering from toothache.

SEC. 1768. Board of examiners: their Appointment and Terms.—The board of examiners shall consist of six practitioners of dentistry, of acknowledged ability in the profession, to be appointed by the Governor. The board shall continue to be divided into three classes with two members each, one of which classes shall go out of office each succeeding year ; and the Governor shall annually appoint the successors of each class, as it goes out, for the term of three years. He shall make the appointments in each case from four persons who shall be nominated by the Virginia State Dental Association, and reside in different sections of the State. All vacancies for unexpired terms shall be filled by the Governor on nominations made by the board. If no nominations be made by the said association or board as the case may be, or the nominations made be not approved by the Governor, he shall appoint such persons as he may deem fit.

SEC. 1769. Their Duties.—It shall be the duty of this board—

*First.* MEETINGS.—To meet annually at the time and place of meeting of the Virginia State Dental Association, or at such other time and place as the board shall agree upon, to conduct the examination of applicants.

They shall also meet for the same purpose at the call of any four members of the board, at such time and place as may be designated by said members. Thirty day's notice of the meetings shall be given by advertising in at least two of the daily papers published in the State.

*Second.* EXAMINATION OF APPLICANTS, ETC.—To grant a certificate of ability to practice dentistry to all applicants who undergo a satisfactory examination, and receive at least four affirmative votes ; which certificate shall be signed by the members of the board, and be stamped with a suitable seal (which they may adopt).

*Third.* REGISTRY.—To keep a book in which shall be registered the name and qualification (as far as practicable) of every person to whom such certificate is granted.

SEC. 1770. Transcripts from Record Book, Evidence.—The book so kept shall be a book of record, and transcripts from it, certified by the officer who has it in keeping, with the seal of the board affixed, shall be evidence in any court of this State

SEC. 1771. Quorum.—Four members of the board shall constitute a quorum ; and should a quorum not be present on any day appointed for their meeting, those present may adjourn from time to time until a quorum be present.

SEC. 1772. Penalties.—Any person who shall, in violation of this chapter, practice dentistry in this State, shall, on conviction thereof, be fined not less than fifty nor more than two hundred dollars, and shall not be entitled to any fee for service rendered; and if a fee shall have been paid, the patient may recover back the same.

SEC. 1773. Disposition of Fines.—All fines collected under this chapter shall go to the public school fund of the county or corporation in which the prosecution is had.

SEC. 1774. Dentists Required to Register.—*Every person practicing dentistry in the Commonwealth of Virginia, at the time of the passage of this act, shall register his name and post-office address, together with the name of the college from which he is a graduate, or the length of time he has been practicing in this Commonwealth, with the board of examiners before renewing his license, and it shall be the duty of the board to issue to each person so registering a certificate of registration stamped with the seal of the board, but no fee shall be collected by the board from persons so registering.*

SEC. 1775. Fees from Applicants.—To provide a fund to carry out the provisions of section seventeen hundred and sixty-nine, it shall be the duty of said board to collect from those who appear before them for examination the sum of ten dollars each.

